# Current gaps in knowledge in inherited arrhythmia syndromes

**DOI:** 10.1007/s12471-023-01797-w

**Published:** 2023-07-06

**Authors:** Puck J. Peltenburg, Lia Crotti, Thomas M. Roston, Christian van der Werf

**Affiliations:** 1 Heart Centre, Amsterdam University Medical Centres, Department of Clinical and Experimental Cardiology, Amsterdam Cardiovascular Sciences, University of Amsterdam, Amsterdam, The Netherlands; 2 Department of Paediatric Cardiology, Emma Children’s Hospital, Amsterdam University Medical Centres, University of Amsterdam, Amsterdam, The Netherlands; 3 Department of Cardiology, IRCCS Istituto Auxologico Italiano, Department of Cardiovascular, Neural and Metabolic Sciences, Ospedale San Luca, Milan, Italy; 4 Department of Medicine and Surgery, University of Milano-Bicocca, Milan, Italy; 5 Centre for Cardiovascular Innovation, Division of Cardiology, University of British Columbia, Vancouver, Canada

**Keywords:** Brugada syndrome, Congenital long QT syndrome, Catecholaminergic polymorphic ventricular tachycardia

## Abstract

The 3 most common inherited arrhythmia syndromes—Brugada syndrome, congenital long QT syndrome and catecholaminergic polymorphic ventricular tachycardia—were initially described in the previous century. Since then, research has evolved, which has enabled us to identify patients prior to the onset of potentially life-threatening symptoms. However, there are significant gaps in knowledge that complicate clinical management of these patients today. With this review paper, we aim to highlight the most important knowledge gaps in clinical research of these inherited arrhythmia syndromes.

## Introduction

The first case reports of the 3 most common inherited arrhythmia syndromes—Brugada syndrome (BrS), congenital long QT syndrome (LQTS) and catecholaminergic polymorphic ventricular tachycardia (CPVT)—were published several decades ago [1–3]. These patients experienced a high rate of syncope, sudden cardiac arrest (SCA) or sudden cardiac death (SCD). Since then, research has evolved to the point where various genes have been implicated and numerous diagnostic tests are used to unmask the phenotype and predict risk. Over time, genetic cascade screening, i.e. screening of a variant associated with a disease in the family of a patient, has led to the steep increase of identification of asymptomatic affected family members for all 3 syndromes. These individuals are generally at significantly lower risk for lethal arrhythmias compared with the proband cases that were initially described and therefore require a different, personalised approach.

In an ideal scenario, one could accurately diagnose a patient with an inherited arrhythmia syndrome early in life, precisely predict who is at greatest risk for life-threatening events and intervene immediately and only if needed with medical and procedural therapies that are highly effective and well tolerated. However, important gaps in our knowledge of these conditions remain and a gold standard for the diagnoses is lacking, which largely preclude such a confident approach. The aim of this paper is to summarise the main limitations of our current clinical knowledge of these rare inherited arrhythmia syndromes.

## Brugada syndrome

**Fig. 1:**
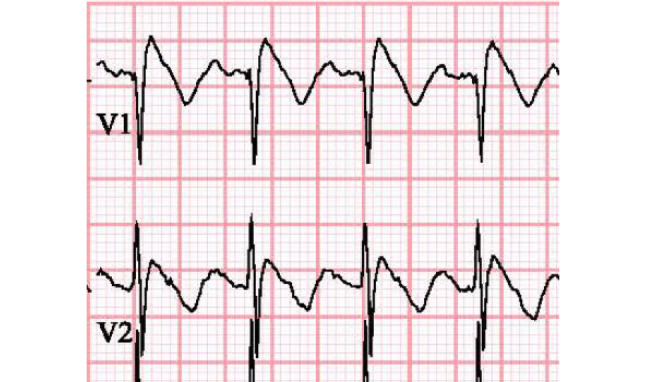
Electrocardiogram of 9‑year-old boy with *SCN5A* variant recorded during fever showing diagnostic type 1 Brugada pattern in precordial leads

Patients with BrS have an abnormal repolarisation pattern in the right precordial leads of the electrocardiogram (ECG) (Fig. 1), which can lead to polymorphic VT, especially during circumstances of high vagal tone, high fever or iatrogenic sodium channel blockade. Throughout the years after its first description in 1992 [3], variable diagnostic criteria and tests have been developed and applied. This has led to the identification of many patients with potential BrS, who are often presumed to be at significant risk for lethal arrhythmias. This is a heterogeneous patient population that all technically carry the same diagnosis of BrS (albeit dependent on the diagnostic criteria applied, as described later), but amongst whom the risk of life-threatening events is also highly heterogeneous. In addition, for BrS patients, in contrast to LQTS and CPVT, effective therapy is mainly limited to implantable cardioverter-defibrillator (ICD) use, which is associated with many lifelong complications, especially in young patients.

### Diagnosis

In 2002, the first expert consensus panel concluded that BrS should be diagnosed in patients with a spontaneous type 1 ECG pattern and in individuals with ECG changes suggestive of BrS that turn into a type 1 ECG pattern during drug challenge [4]. The inclusion of a type 1 pattern after a drug challenge was adopted in a subsequent consensus agreement in 2013 and in the 2015 European Society of Cardiology (ESC) Guidelines for the management of patients with ventricular arrhythmias and the prevention of sudden cardiac death [5, 6]. In 2016, the first concerns regarding overdiagnosis when using the drug challenge were described. A drug challenge provoked a type 1 ECG pattern in ~4% of healthy volunteers [7]. Thereafter, the Shanghai criteria were developed to improve the specificity of a BrS diagnosis [8], thereby also taking into account inferential weighted factors from clinical and family history and genetic test results, which have now been adopted in the 2022 ESC Guidelines for patients with ventricular arrhythmias [9]. With these stringent criteria, a patient with a type 1 ECG pattern after a drug challenge should only be diagnosed with ‘probable’ or ‘definite’ BrS in the presence of, amongst others, a *SCN5A* gene variant—which encodes for the cardiac sodium channel and is most commonly associated with BrS—, a history of arrhythmic syncope or documented VT/ventricular fibrillation, or a family history of BrS.

Therefore, many patients would now be considered as having only ‘possible’ BrS according to the latest Shanghai criteria (i.e. based on a positive drug challenge in the absence of additional diagnostic criteria, and *SCN5A* variant carrying family members without ECG abnormalities). A possible BrS diagnosis is, understandably, accompanied by uncertainty and anxiety for many patients [10], in whom the certainty of carrying a life-threatening diagnosis has been amended. However, for some of these patients, despite having a low overall risk of symptoms (estimated 0.5% per year for arrhythmic events), the first sign or symptom might still be lethal [11]. Furthermore, as the type 1 pattern is known to be variable over time [12], these patients may still develop a spontaneous type 1 pattern during follow-up. Future studies should focus on this patient group in particular, to refine the diagnosis and investigate how to best counsel, follow-up and genetically test these patients and their family members.

### Risk stratification

BrS was first described in 3 patients with an SCA and the typical type 1 pattern on their ECGs [3]. Since then, many asymptomatic patients with this same ECG pattern at rest or provoked by a drug challenge were identified. Their risk of events, however, differs from that of the patients initially described. Previous symptoms and a spontaneous type 1 ECG pattern are independently and reproducibly associated with future events [13–15].

A recent large study on risk stratification showed that, in addition to symptoms and a spontaneous type 1 ECG pattern, early repolarisation and a type 1 ECG pattern in peripheral leads were independently associated with future events [16]. As BrS is a familial disease, mainly genetic factors may be of interest for future risk stratification studies [17]. However, the proportion of *SCN5A* carriers is low [18], and the syndrome seems to be polygenic: multiple single nucleotide polymorphisms add to the risk of developing the BrS phenotype [19]. Future studies should mainly focus on improving risk stratification of asymptomatic BrS patients to identify who might benefit from treatment.

### Treatment

Treatment with an ICD is currently reserved for BrS patients who are at high risk for events, thus those with an SCA or arrhythmic syncope [9]. Isoproterenol can be used in patients suffering from an electrical storm [9].

In 2011, ablation of the right ventricular outflow tract epicardium was described as a treatment to prevent ventricular arrhythmias in symptomatic patients [20]. During 28-month follow-up, recurrence of sustained ventricular arrhythmia occurred in 17.6% of cases, while complications were seen in 9.3% [21]. These numbers underline the need for further studies regarding the long-term effect of ablation in the prevention of symptoms and, as a secondary endpoint, the ECG characteristics. Furthermore, since this invasive procedure comes with a risk of complications, ablation is not recommended for the asymptomatic population and should be approached with extreme caution [9].

Quinidine seems to be promising in preventing arrhythmias but is associated with a high risk of side effects [22]. Some experts in the field advocate the use of quinidine as preventive treatment for asymptomatic BrS patients [10], and low-dose quinidine has been shown to be associated with a lower rate of side effects [23]. Long-term prevention of arrhythmias with low-dose quinidine in the asymptomatic population, however, still needs to be established.

## Catecholaminergic polymorphic ventricular tachycardia

**Fig. 2:**
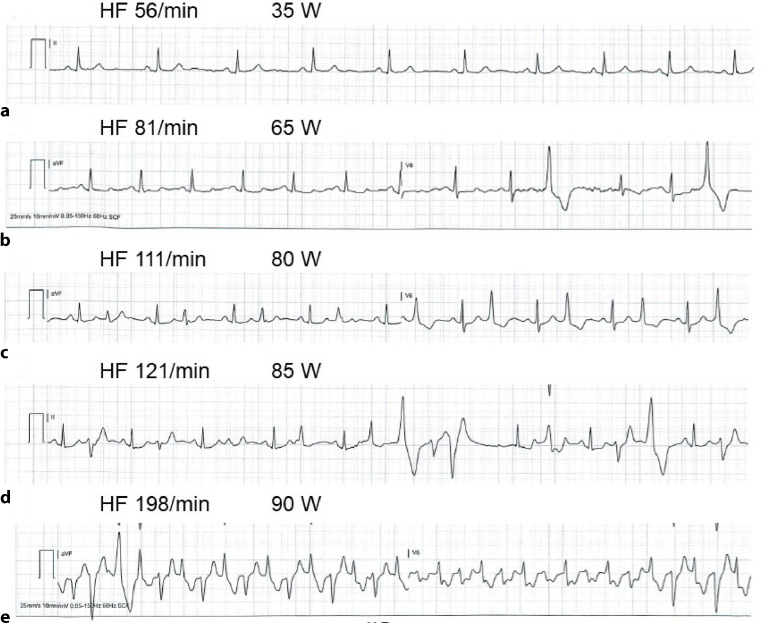
Exercise stress test of patient with catecholaminergic polymorphic ventricular tachycardia showing ventricular arrhythmia that increased in severity as workload attenuated and resolved after cessation of exercise. **a** Normal sinus rhythm without premature ventricular complexes (PVCs), **b** isolated PVCs, **c** PVCs in bigeminy, **d** polymorphic couplet and triplet following PVCs in bigeminy, and **e** typical bidirectional ventricular tachycardia at peak exercise. *HF* heart frequency

CPVT is characterised by stress-induced polymorphic and bidirectional ventricular arrhythmias (Fig. 2). The diagnosis is made when exercise stress testing (EST) unmasks the arrhythmic phenotype but cardiac imaging reveals no evidence of structural heart disease [5]. Most cases are caused by variants in *RYR2*, a gene that codes for the component proteins of the large homotetrameric cardiac ryanodine receptor, which controls calcium release from the sarcoplasmic reticulum.

Minimum baseline treatment is a non-selective beta-blocker, with effective adjunct therapies such as flecainide and left cardiac sympathetic denervation (LCSD) added on if needed [5]. ICD therapy may seem to be a logical strategy to prevent SCD, but its benefits may be offset by a high risk of inappropriate therapies related to the catecholamine-stimulating nature of painful shocks or ineffective therapies in the case of a shock elicited by triggered activity (bidirectional or polymorphic VT) [24, 25].

Comprehensive reviews of the mechanism, phenotype and treatment of CPVT are available elsewhere [26]. These works summarise the amazing progress that has been made in this condition over the past 30 years. However, many unanswered questions related to CPVT remain, and the greatest focus here will be placed on those most critical to immediate patient care.

### Diagnosis

The definition of CPVT, established by an expert consensus statement a decade ago [5], may be overly inclusive, with refinement anticipated in future iterations. The existing requirement of stress-induced polymorphic and/or bidirectional ventricular arrhythmias in the absence of structural heart disease, including coronary artery disease in those over 40 years of age, means a broad range of patients have at least possible CPVT.

This has led to studies suggesting the existence of 2 forms of the condition [27]. The classical type of CPVT affects children and adolescents and is characterised by a moderately reproducible EST phenotype at predictable heart rate thresholds and in the presence of a damaging *RYR2* variant [28]. In contrast, other forms of stress-induced complex arrhythmias have been described that usually occur in older individuals and in the setting of concurrent ambient ventricular arrhythmias [27]. While this latter phenotype technically meets CPVT diagnostic criteria, it is very likely a different disease entity based on the aforementioned findings.

The expert opinion-based diagnostic score card for CPVT, designed predominantly for novel variant adjudication, incorporates contemporary phenotypic characteristics that support the CPVT diagnosis [29]. These include factors that upgrade (e.g. younger age, gene positivity, family history) or downgrade (ambient ventricular ectopy, ischaemic heart disease, longer QT interval) the likelihood of CPVT, akin to the LQTS risk diagnostic score for LQTS and the Shanghai criteria for BrS. Future guideline-based definitions are likely to refine the diagnosis of CPVT in a similar manner, but for now, some CPVT populations—in particular those with large proportions of non-genotyped or genotype-negative patients—may be too heterogeneous to make highly accurate estimates of disease risk and treatment efficacy.

### Risk stratification

A remaining issue central to both the diagnosis and treatment of CPVT rests on the sensitivity and specificity of the EST for diagnosing and risk-stratifying the condition. Traditionally, EST protocols used mainly for ischaemic heart disease, such as the Bruce protocol, were also used for CPVT out of convenience. It appears, however, that the severity of ventricular arrhythmia is only moderately repeatable, especially in patients taking medication, potentially reflecting reduced therapeutic adherence [28].

Recently, a novel ‘burst’ EST protocol has been described in a small number of patients as being capable of better unmasking CPVT arrhythmias [30]. This test involves a sudden sprint rather than gradually graded exercise. If this protocol is indeed more sensitive, it could also assist with optimising therapeutic decision-making. Similarly, other electrocardiographic modalities, such as prolonged 12-lead Holter recording or patch monitoring, are generally not used to make the diagnosis or titrate medical therapy. While such tests may seem simple to implement, it is important to appreciate that broader ascertainment of potential CPVT cases through additional testing may lead to overdiagnosis or misdiagnosis. The roles of such testing need to be better studied.

### Treatment

Evidence-based risk stratification and therapeutic escalation also remain challenging in CPVT. Flecainide and LCSD are now established adjunctive therapies to non-selective beta-blockers, however, indications for and timing of these interventions are unclear. Likewise, by following the recommended use of flecainide only after maximising the beta-blocker dose, a group of patients who would benefit from an empiric multi-targeted approach with combination of flecainide and beta-blocker is probably overlooked.

Furthermore, the number of breakthrough events despite prescription of optimal medical therapy is significant and often related to non-adherence. Thus, there is a need for properly understanding the true rate of non-adherence and the reasons thereof. Subsequently, LCSD is certainly an excellent option, but whether it should be instituted before, with or after flecainide has not been studied, and whether asymptomatic patients with ongoing ventricular ectopy should undergo this invasive procedure is also not clear.

An even greater uncertainty lies in identifying the small group of patients in whom an ICD is of greater benefit than risk. This is a critical problem since practices clearly vary across the world [25, 31], and designing rigorous studies is complicated by the fact that only patients with ICDs can meet endpoints involving appropriate shocks and programming may play a large role in both appropriate and inappropriate shocks.

Finally, while a substantial focus has been placed on the highest-risk patients, relatively little is known about the management of minimally affected or asymptomatic patients. Unlike in BrS and LQTS, intentional non-treatment of phenotype-negative CPVT is currently not a broadly accepted practice. However, a lower-risk group of genotype-positive patients certainly exists, as evidenced by large founder populations in the Netherlands and on the Canary Islands [32, 33], suggesting that not all asymptomatic, phenotype-negative patients benefit from being on beta-blockers.

## Long QT syndrome

In LQTS, arrhythmia symptoms typically occur during emotional triggers, sleep or exercise, depending on the genetic variant causing the syndrome. It is mainly diagnosed in patients with a prolonged heart rate-corrected QT interval (QTc) on their baseline ECG. The higher incidence has enabled the identification of some clear genotype-specific risk groups in which treatment should be intensified—with avoidance of QT-prolonging drugs, beta-blockers, LCSD, ICD implantation—to reduce the high risk of a recurrent life-threatening event [34]. However, knowledge gaps similar to those described for BrS and CPVT currently complicate the clinical management of LQTS patients.

### Diagnosis

LQTS is diagnosed in patients with a QTc of ≥ 480 ms on the baseline ECG in the absence of secondary causes of QT prolongation or when the LQTS risk score—taking ECG findings, clinical and genetic findings, and family history into account—is > 3 points [9, 35]. The role of EST has been well established in the diagnosis of LQTS, and indeed, 1 point in the LQTS risk score is assigned when the QTc is ≥ 480 ms in the fourth minute of the recovery phase.

The role of long-term ECG monitoring—mainly 24-hour, 12-lead Holter recording—is not yet well established. In fact, it is well known that the QTc exhibits variability during the day [36], and the presence of a QTc of 480 ms on a Holter recording therefore does not have the same value in terms of diagnosis as the same QTc observed on a baseline ECG. However, definitive diagnostic criteria for Holter-derived QTc are not available. This is an especially important issue, as smartwatches and smartphone-enabled electrodes that are useful for QTc assessment [37] and can even accurately automatically calculate the QTc [38] are currently becoming publicly available. This will most likely lead to an increase of borderline QTc measurements, for which clear cutoff values and diagnostic criteria are needed.

### Risk stratification

Some patient characteristics are well recognised as risk factors for future events, such as QTc prolongation, ECG signs of electrical instability (i.e. T‑wave alternans), the presence of a pathogenic variant [39] and a specific variant in some cases [40], and the protein region of the disease-causing variant or its functional consequences [41]. LQTS is, without any doubt, the disease for which there is the most expertise in using genetic data for risk stratification. However, a number of gaps are still present.

Risk stratification is now possible in patients with LQT1, LQT2 and LQT3 [42], caused by variants in the *KCNQ1, KCNH2* and *SCN5A* genes, respectively. However, the risk stratification is based on the 5‑year arrhythmic risk of LQTS patients without anti-arrhythmic therapy. In LQTS, effective therapies are available that clearly reduce arrhythmic risk. This should be taken into account when estimating the 5‑year arrhythmic risk upon which a decision to implant an ICD for primary prevention is based. Accurate risk stratification tools are lacking for genotype-negative subjects, patients with rarer genetic subtypes, patients with overlapping phenotypes, i.e. BrS, LQTS and cardiac conduction disease [43], and patients with other cardiac comorbidities (i.e. LQTS and coexisting cardiomyopathy or LQTS and myocardial infarction). The role of modifier genes in the arrhythmic risk has been described [44] but has not been implemented in clinical practice.

### Treatment

**Fig. 3:**
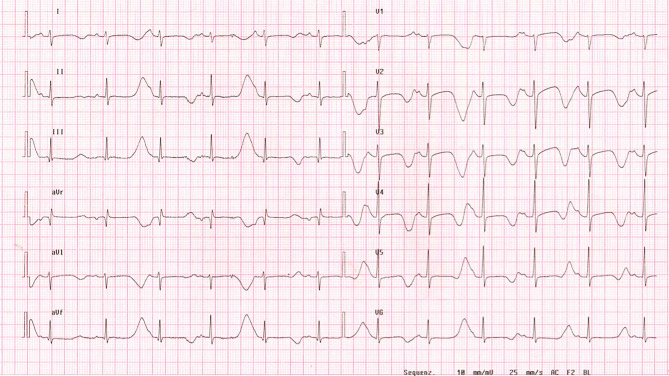
Twelve-lead electrocardiogram of 9‑year-old girl with long QT syndrome (*CALM1* variant) and history of multiple cardiac arrests, showing severely prolonged heart rate-corrected QT interval (QTc) and T‑wave alternans

Evidence-based treatment regimens remain especially ill-defined for patients with life-threatening arrhythmias in the first year of life. These are patients at extremely high risk for the recurrence of events [45]. Furthermore, conventional therapies frequently fail to prevent the recurrence of major cardiac events in this population [46]. ICDs are also not designed for such young children and increase the risk of complications related to device implantation and replacement. Frequently, these young patients have specific high-risk genetic subtypes, such as *CALM* gene variants (Fig. 3; [39]) or *TRDN* gene variants [47], Timothy syndrome (LQTS type 8) or autosomal recessive LQTS (Jervell and Lange-Nielsen syndrome) [48]. Therefore, more gene-specific therapies, in addition to mexiletine for patients with LQTS types 2 [49] and 3 [50], are needed.

On the other end of the spectrum are asymptomatic LQTS patients with a normal baseline QTc and therefore a low perceived risk of symptoms. This group should be studied further, with a long-term follow-up in order to distinguish patients at risk for symptoms—who could thus potentially profit from beta-blocker treatment—from those who remain asymptomatic and can be left untreated.

## Conclusion

Our contemporary understanding of the 3 main forms of inherited arrhythmia syndromes represents an incredible feat of modern medicine and research. Nevertheless, important gaps in knowledge remain and pose major barriers to the optimal health and well-being of patients affected by these disorders. History has proven that the development of diagnostic criteria is difficult and subject to changes as new data emerge.

On the one hand, the number of patients identified prior to the occurrence of symptoms—such as genotype-positive, phenotype-negative patients—has increased over time and will continue to rise in the future. This is a group of patients in whom the natural history of the condition is often unknown, and thus we need more data on risk stratification to provide appropriate patient-specific therapy and follow-up regimens. On the other hand, there are clearly symptomatic patients, who have suffered from an arrhythmic syncope or survived an SCA. In this population, there is a need for optimisation of medical therapy.

Short-term advancements may come in the form of repurposed medications, better selection of patients who will benefit from an ICD or a combination of both, while awaiting the development and implementation of new medications, including potentially gene therapy. The potential complications of an ICD should be outweighed by the beneficial effects in terms of SCD prevention in all conditions.

To fill all gaps of knowledge described in this paper, large cohort studies are needed. Therefore, continuous collaborations between clinicians and researchers worldwide are of the utmost importance to advance in this field.

## References

[1] Horan MV, Venables AW (1962). Paroxysmal tachycardia with episodic unconsciousness. Arch Dis Child.

[2] Langslet A, Sorland SJ (1975). Surdocardiac syndrome of Jervell and Lange-Nielsen, with prolonged QT interval present at birth, and severe anaemia and syncopal attacks in childhood. Br Heart J.

[3] Brugada P, Brugada J (1992). Right bundle branch block, persistent ST segment elevation and sudden cardiac death: a distinct clinical and electrocardiographic syndrome. A multicenter report. J Am Coll Cardiol.

[4] Wilde AA, Antzelevitch C, Borggrefe M (2002). Proposed diagnostic criteria for the Brugada syndrome: consensus report. Circulation.

[5] Priori SG, Wilde AA, Horie M (2013). HRS/EHRA/APHRS expert consensus statement on the diagnosis and management of patients with inherited primary arrhythmia syndromes: document endorsed by HRS, EHRA, and APHRS in May 2013 and by ACCF, AHA, PACES, and AEPC in June 2013. Heart Rhythm.

[6] Priori SG, Blomstrom-Lundqvist C, Mazzanti A (2015). 2015 ESC guidelines for the management of patients with ventricular arrhythmias and the prevention of sudden cardiac death: the task force for the management of patients with ventricular arrhythmias and the prevention of sudden cardiac death of the European society of cardiology (ESC). Endorsed by: association for European paediatric and congenital cardiology (AEPC). Eur Heart J.

[7] Hasdemir C, Payzin S, Kocabas U (2015). High prevalence of concealed Brugada syndrome in patients with atrioventricular nodal reentrant tachycardia. Heart Rhythm.

[8] Antzelevitch C, Yan GX, Ackerman MJ (2016). J-wave syndromes expert consensus conference report: emerging concepts and gaps in knowledge. J Arrhythm.

[9] Zeppenfeld K, Tfelt-Hansen J, de Riva M (2022). 2022 ESC guidelines for the management of patients with ventricular arrhythmias and the prevention of sudden cardiac death. Eur Heart J.

[10] Viskin S, Hochstadt A, Schwartz AL, Rosso R (2021). Will I die from Brugada syndrome?: the rumination of risk stratification. JACC Clin Electrophysiol.

[11] Milman A, Andorin A, Gourraud JB (2018). Profile of patients with Brugada syndrome presenting with their first documented arrhythmic event: data from the survey on arrhythmic events in BRUgada syndrome (SABRUS). Heart Rhythm.

[12] Veltmann C, Schimpf R, Echternach C (2006). A prospective study on spontaneous fluctuations between diagnostic and non-diagnostic ECGs in Brugada syndrome: implications for correct phenotyping and risk stratification. Eur Heart J.

[13] Sieira J, Conte G, Ciconte G (2017). A score model to predict risk of events in patients with Brugada syndrome. Eur Heart J.

[14] Probst V, Goronflot T, Anys S (2021). Robustness and relevance of predictive score in sudden cardiac death for patients with Brugada syndrome. Eur Heart J.

[15] Kawada S, Morita H, Antzelevitch C (2018). Shanghai score system for diagnosis of Brugada syndrome: validation of the score system and system and reclassification of the patients. JACC Clin Electrophysiol.

[16] Honarbakhsh S, Providencia R, Garcia-Hernandez J (2021). A primary prevention clinical risk score model for patients with Brugada syndrome (BRUGADA-RISK). JACC Clin Electrophysiol.

[17] Ishikawa T, Kimoto H, Mishima H (2021). Functionally validated SCN5A variants allow interpretation of pathogenicity and prediction of lethal events in Brugada syndrome. Eur Heart J.

[18] Schulze-Bahr E, Eckardt L, Breithardt G (2003). Sodium channel gene (SCN5A) mutations in 44 index patients with Brugada syndrome: different incidences in familial and sporadic disease. Hum Mutat.

[19] Barc J, Tadros R, Glinge C (2022). Genome-wide association analyses identify new Brugada syndrome risk loci and highlight a new mechanism of sodium channel regulation in disease susceptibility. Nat Genet.

[20] Nademanee K, Veerakul G, Chandanamattha P (2011). Prevention of ventricular fibrillation episodes in Brugada syndrome by catheter ablation over the anterior right ventricular outflow tract epicardium. Circulation.

[21] Kotake Y, Barua S, Kazi S (2022). Efficacy and safety of catheter ablation for Brugada syndrome: an updated systematic review. Clin Res Cardiol.

[22] Andorin A, Gourraud JB, Mansourati J (2017). The QUIDAM study: hydroquinidine therapy for the management of Brugada syndrome patients at high arrhythmic risk. Heart Rhythm.

[23] Mazzanti A, Tenuta E, Marino M, Pagan E, Morini M, Memmi M (2019). Efficacy and limitations of quinidine in patients with Brugada syndrome. Circ Arrhythm Electrophysiol.

[24] Roston TM, Jones K, Hawkins NM (2018). Implantable cardioverter-defibrillator use in catecholaminergic polymorphic ventricular tachycardia: a systematic review. Heart Rhythm.

[25] Van der Werf C, Lieve KV, Bos JM (2019). Implantable cardioverter-defibrillators in previously undiagnosed patients with catecholaminergic polymorphic ventricular tachycardia resuscitated from sudden cardiac arrest. Eur Heart J.

[26] Priori SG, Mazzanti A, Santiago DJ (2021). Precision medicine in catecholaminergic polymorphic ventricular tachycardia: JACC focus seminar 5/5. J Am Coll Cardiol.

[27] Sy RW, Gollob MH, Klein GJ (2011). Arrhythmia characterization and long-term outcomes in catecholaminergic polymorphic ventricular tachycardia. Heart Rhythm.

[28] Peltenburg PJ, Pultoo SNJ, Tobert KE (2023). Repeatability of ventricular arrhythmia characteristics on the exercise-stress test in RYR2-mediated catecholaminergic polymorphic ventricular tachycardia. Europace.

[29] Giudicessi JR, Lieve KVV, Rohatgi RK (2019). Assessment and validation of a phenotype-enhanced variant classification framework to promote or demote RYR2 missense variants of uncertain significance. Circ Genom Precis Med.

[30] Roston TM, Kallas D, Davies B (2021). Burst exercise testing can unmask arrhythmias in patients with incompletely penetrant catecholaminergic polymorphic ventricular tachycardia. JACC Clin Electrophysiol.

[31] Roston TM, Vinocur JM, Maginot KR (2015). Catecholaminergic polymorphic ventricular tachycardia in children: analysis of therapeutic strategies and outcomes from an international multicenter registry. Circ Arrhythm Electrophysiol.

[32] Van der Werf C, Nederend I, Hofman N (2012). Familial evaluation in catecholaminergic polymorphic ventricular tachycardia: disease penetrance and expression in cardiac ryanodine receptor mutation-carrying relatives. Circ Arrhythm Electrophysiol.

[33] Wanguemert F, Bosch Calero C, Perez C (2015). Clinical and molecular characterization of a cardiac ryanodine receptor founder mutation causing catecholaminergic polymorphic ventricular tachycardia. Heart Rhythm.

[34] Schwartz PJ, Crotti L, Insolia R (2012). Long-QT syndrome: from genetics to management. Circ Arrhythm Electrophysiol.

[35] Schwartz PJ, Crotti L (2011). QTc behavior during exercise and genetic testing for the long-QT syndrome. Circulation.

[36] Molnar J, Zhang F, Weiss J, Ehlert FA, Rosenthal JE (1996). Diurnal pattern of QTc interval: how long is prolonged? Possible relation to circadian triggers of cardiovascular events. J Am Coll Cardiol.

[37] Bergeman AT, Pultoo SNJ, Winter MM (2022). Accuracy of mobile 6-lead electrocardiogram device for assessment of QT interval: a prospective validation study. Neth Heart J.

[38] Giudicessi JR, Schram M, Bos JM (2021). Artificial intelligence-enabled assessment of the heart rate corrected QT interval using a mobile electrocardiogram device. Circulation.

[39] Crotti L, Johnson CN, Graf E (2013). Calmodulin mutations associated with recurrent cardiac arrest in infants. Circulation.

[40] Crotti L, Spazzolini C, Schwartz PJ (2007). The common long-QT syndrome mutation KCNQ1/A341V causes unusually severe clinical manifestations in patients with different ethnic backgrounds: toward a mutation-specific risk stratification. Circulation.

[41] Crotti L (2023). From gene-specific to function-specific risk stratification in long QT syndrome type 2: implications for clinical management. Europace.

[42] Mazzanti A, Trancuccio A, Kukavica D (2022). Independent validation and clinical implications of the risk prediction model for long QT syndrome (1-2-3-LQTS-risk). Europace.

[43] Makita N, Behr E, Shimizu W (2008). The E1784K mutation in SCN5A is associated with mixed clinical phenotype of type 3 long QT syndrome. J Clin Invest.

[44] Schwartz PJ, Crotti L, George AL (2018). Modifier genes for sudden cardiac death. Eur Heart J.

[45] Spazzolini C, Mullally J, Moss AJ (2009). Clinical implications for patients with long QT syndrome who experience a cardiac event during infancy. J Am Coll Cardiol.

[46] Moore JP, Gallotti RG, Shannon KM (2020). Genotype predicts outcomes in fetuses and neonates with severe congenital long QT syndrome. JACC Clin Electrophysiol.

[47] Clemens DJ, Tester DJ, Giudicessi JR (2019). International triadin knockout syndrome registry. Circ Genom Precis Med.

[48] Schwartz PJ, Spazzolini C, Crotti L (2006). The Jervell and Lange-Nielsen syndrome: natural history, molecular basis, and clinical outcome. Circulation.

[49] Bos JM, Crotti L, Rohatgi RK (2019). Mexiletine shortens the QT interval in patients with potassium channel-mediated type 2 long QT syndrome. Circ Arrhythm Electrophysiol.

[50] Mazzanti A, Maragna R, Faragli A (2016). Gene-specific therapy with mexiletine reduces arrhythmic events in patients with long QT syndrome type 3. J Am Coll Cardiol.

